# Boolean implication networks derived from large scale, whole genome microarray datasets

**DOI:** 10.1186/gb-2008-9-10-r157

**Published:** 2008-10-30

**Authors:** Debashis Sahoo, David L Dill, Andrew J Gentles, Robert Tibshirani, Sylvia K Plevritis

**Affiliations:** 1 Department of Electrical Engineering, Stanford University, Stanford, CA 94305, USA; 2 Department of Computer Science, Stanford University, Stanford, CA 94305, USA; 3 Department of Radiology, Stanford University, Stanford, CA 94305, USA; 4 Department of Health Research and Policy and Department of Statistics, Stanford University, Stanford, CA 94305, USA

## Abstract

We describe a method for extracting Boolean implications (if-then relationships) in very large amounts of gene expression microarray data. A meta-analysis of data from thousands of microarrays for humans, mice, and fruit flies finds millions of implication relationships between genes that would be missed by other methods. These relationships capture gender differences, tissue differences, development, and differentiation. New relationships are discovered that are preserved across all three species.

## Abstract 2

A method for analysis of microarray data is presented that extracts statistically significant Boolean implication relationships between pairs of genes.

## Background

A large and exponentially growing volume of gene expression data from microarrays is now available publicly. Since the quantity of data from around the world dwarfs the output of any individual laboratory, there are opportunities for mining these data that can yield insights that would not be apparent from smaller, less diverse data sets. Consequently, numerous approaches for extracting large networks of relationships from large amounts of public-domain gene expression data have been used. Almost all of this work constructs networks of pairwise relationships between genes, indicating that the genes are co-expressed [1-5]. Co-expression is a symmetric relationship between a gene pair, because if A is related to B, then B is related to A. Many of these methods are based on showing that the expression of two genes has a coefficient of correlation exceeding some threshold.

We propose a new approach to identify a larger set of relationships between gene pairs across the whole genome using data from thousands of microarray experiments. We first classify the expression level of each gene on each array as 'low' or 'high' relative to an automatically determined threshold that is derived individually for each gene. We then identify all Boolean implications between pairs of genes. An implication is an if-then rule, such as 'if gene A's expression level is high, then gene B's expression level is almost always low', or more concisely, 'A high implies B low', written 'A high ⇒ B low'.

In general, Boolean implications are asymmetric: 'A high ⇒ B high' may hold for the data without 'B high ⇒ A high' holding. However, it is also possible that both of these implications hold, in which case A and B are said to be 'Boolean equivalent'. Booleanequivalence is a symmetric relationship. Equivalent genes are usually strongly correlated as well. A second kind of symmetric relationship occurs when A high ⇒ B low and B high ⇒ A low. In this case, the expression levels of A and B are usually strongly negatively correlated, and genes A and B are said to be 'opposite'. In total, six possible Boolean relationships are identified: two symmetric (equivalent and opposite) and four asymmetric (A low ⇒ B low, A low ⇒ B high, A high ⇒ B low, B high ⇒ A high). Below, 'symmetric relationship' means a Boolean equivalence or opposite relationship; 'asymmetric relationship' means any of the four kinds of implications, when the converse relationship does not hold; and 'relationship' means any of the two symmetric or four asymmetric relationships.

The set of Boolean implications is a labeled directed graph, where the vertices are genes (more precisely, Affymetrix probesets for genes, in our data) and the edges are implications, labeled with the implication type. We call this graph the Boolean implication network. Networks based on symmetric relationships are undirected graphs.

It is important to understand that a Boolean implication is an empirically observed invariant on the expression levels of two genes and does not necessarily imply any causality. One way to understand the biological significance of a Boolean implication is to consider the sets of arrays where the two genes are expressed at a high level. The asymmetric Boolean implication A high ⇒ B high means that 'the set of arrays where A is high is a subset of the set of arrays where B is high'. For example, this may occur when gene B is specific to a particular cell type, and gene A is specific to a subclass of those cells. Alternatively, this implication can be the result of a regulatory relationship, so A high ⇒ B high could hold because A is one of several transcription factors that increases expression of B, or because B is a transcription factor that increases expression of A only in the presence of one or more cofactors. On the other hand, the asymmetric Boolean implication A high ⇒ B low means that A and B are rarely high on the same array - the genes are 'mutually exclusive'. A possible explanation for this is that A and B are specific to distinct cell types (for example, brain versus prostate), or it could be that A represses B or *vice versa*.

Boolean implications capture many more relationships that are overlooked by existing methods that scale to large amounts of data, which generally find only symmetric relationships. There may be a highly significant Boolean implication between genes whose expression is only weakly correlated. The relationships in the resulting network are often biologically meaningful. The network identifies Boolean implications that describe known biological phenomena, as well as many new relationships that can serve to generate new hypotheses. Moreover, many previously unidentified relationships are conserved between humans, mice, and fruit flies.

A meta-analysis was performed on thousands of publicly available microarray datasets on Affymetrix platforms for humans, mice, and fruit flies. This is the first time Boolean implication networks have been applied to the problem of mining large quantities of microarray data. The remainder of this manuscript explains how the networks are constructed from gene-expression microarray datasets, and describes selected Boolean implications that capture important biological phenomena that would be overlooked in gene expression networks based on co-expression. We also discuss related work.

## Results and discussion

### Boolean implications are prevalent in gene expression microarray data

**Figure 1:**
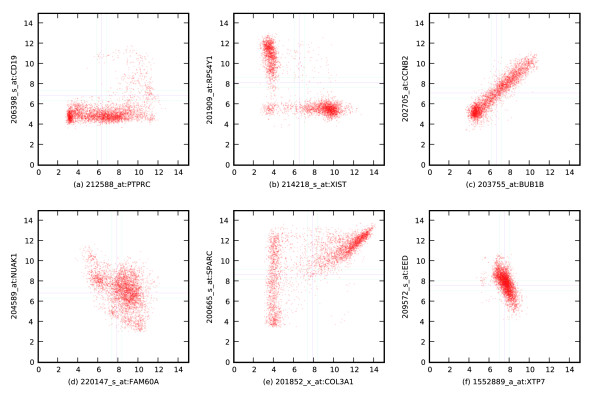
Boolean relationships. Six different types of Boolean relationships between pairs of genes taken from the Affymetrix U133 Plus 2.0 human dataset. Each point in the scatter plot corresponds to a microarray experiment, where the two axes correspond to the expression levels of two genes. There are 4,787 points in each scatter plot. **(a) **PTPRC low ⇒ CD19 low. **(b) **XIST high ⇒ RPS4Y1 low. **(c) **Equivalent relationship between CCNB2 and BUB1B. **(d) **FAM60A low ⇒ NUAK1 high. **(e) **COL3A1 high ⇒ SPARC high. **(f) **Opposite relationship between EED and XTP7.

Boolean implication networks are constructed by finding Boolean implications between pairs of probesets in hundreds or thousands of microarrays belonging to the same platform. The logarithm (base 2) of each expression level is used. To find a Boolean implication between a pair of genes, each probeset is assigned an expression threshold *t *(see Materials and methods). A scatter plot where each point represents gene A's expression versus gene B's expression for a single sample is divided, based on the thresholds, into four quadrants: (A low, B low), (A low, B high), (A high, B low), and (A high, B high). A Boolean implication exists when one or more quadrants is sparsely populated according to a statistical test and there are enough high and low values for each gene (to prevent the discovery of implications that follow from an extreme skew in the distribution of one of the genes). The test produces a score, and a cutoff is chosen for the presence or absence of an implication to obtain an acceptable false discovery rate (FDR; see Materials and methods). To reduce sensitivity to small errors in the choice of *t *and noise in the data, points within an interval around the threshold are ignored (see Materials and methods). A visual examination of the scatter plots is a straightforward way to understand the implications and to check the quality of the results (Figure 1).

There are four possible asymmetric Boolean relationships, each occurring when a particular quadrant is sparse. Figure 1a shows an example low ⇒ low implication; here the quadrant is sparse when PTPRC is low and CD19 high, so PTPRC low ⇒ CD19 low. Figure 1b shows a high ⇒ low implication; here XIST high ⇒ RPS4Y1 low; this relationship was recently identified in a study of the CELSIUS microarray database [6], which annotated microarrays by gender. Figure 1d shows a low ⇒ high implication; here FAM60A low ⇒ NUAK1 high. In this case, when FAM60A expression level is low, NUAK1 expression level is high, but when FAM60A expression level is high, NUAK1 expression level is evenly distributed between high and low. Finally, Figure 1e shows a high ⇒ high implication; here COL3A1 high ⇒ SPARC high. This particular relationship may be viewed as complex, since it involves a combination of multiple types of relationships, including linear and constant. However, from a Boolean perspective, this is a simple and clear logical implication, which is easily detected.

For each of the above asymmetric Boolean implications, there is always a contrapositive Boolean relationship. (The contrapositive is the implication that results from swapping the left-hand and right-hand genes while simultaneously changing low to high and vice versa.) For example, PTPRC low ⇒ CD19 low so CD19 high ⇒ PTPRC high. Similarly, XIST high ⇒ RPS4Y1 low, so RPS4Y1 high ⇒ XIST low; FAM60A low ⇒ NUAK1 high, so NUAK1 low ⇒ FAM60A high; and COL3A1 high ⇒ SPARC high, so SPARC low ⇒ COL3A1 low.

The two possible symmetric Boolean relationships correspond to two sparse diagonally opposed quadrants in a scatter plot. First, the low-high and high-low quadrant can be sparse as shown in Figure 1c, which shows that CCNB2 and BUB1B are equivalent in the human network. Strongly positively correlated genes are almost always equivalent. Alternatively, the low-low and high-high quadrants can be sparse, as shown in Figure 1f, which shows that EED and XTP7 are opposite. Negatively correlated genes are often opposite. An important reason for ignoring points that are close to the low/high threshold is to enable discovery of equivalence and opposite relationships. As is clear in Figure 1c, if points inside the intermediate region were considered, there would be a significant number of points in all four quadrants. Empirically, the interval width of 1 results in the discovery of many equivalent genes. Notice that it is not possible to have both the low-low and high-low quadrants be sparse because that would require the second gene to be always low; similarly, it is not possible for the low-high and low-low quadrants both to be sparse.

We constructed Boolean implication networks for humans, mice, and fruit flies in a meta-analysis of publicly available microarray data. A very large number of Boolean implications were found for each individual species. Approximately 3 billion probeset pairs were checked for possible Boolean implications in the human dataset, of which 208 million were significant implications, even with a stringent requirement for significance (a permutation test yields a FDR of 10^-4^). Similarly, the mouse dataset has 336 million implications out of 2 billion probeset pairs (with an FDR of 6 × 10^-5^), and the fruit fly dataset has 17 million implications out of 196 million probeset pairs (with an FDR of 6 × 10^-6^). Of the 208 million implications in the human dataset, 128 million are high ⇒ low, 38 million are low ⇒ low, 38 million are high ⇒ high, 2 million are low ⇒ high, 1.6 million relationships are equivalences and 0.4 million are opposite.

Table 1 summarizes the number of Boolean relationships found in each dataset. In all cases, Boolean implications of the type high ⇒ low are most common, and opposite relationships are rare. As can be seen from Table 1, in the human data set, 1% of the total Boolean relationships are symmetric, while the remaining 99% are asymmetric. Similarly, in the mouse data set, 1.4% of the total Boolean relationships are symmetric, and 98.6% are asymmetric. However, in the fruit fly dataset 12% of the Boolean relationships are symmetric. The number of low ⇒ low relationships is always the same as the number of high ⇒ high relationships because of contrapositives. One reason for the large number of high ⇒ low relationships is that there are many genes that are specific to particular cell and tissue types, and *n *mutually exclusively expressed genes give rise to *n*(*n *- 1) high ⇒ low relationships.

**Table 1:** Number (in millions) of Boolean relationships in human, mouse and fruit fly datasets

Dataset	Total	Low implies high	High implies how	Low implies how	High implies high	Equivalent	Opposite
Human	208	2	128	38	38	1.6	0.4
Mouse	336	8	208	57.6	57.6	4.1	0.7
Fruit fly	17	0.3	7.3	3.7	3.7	1.9	0.1

In the human dataset, 1% of all Boolean relationships are symmetric (equivalence + opposite) and 99% are asymmetric (low ⇒ low + low ⇒ high + high ⇒ low + high ⇒ high). The mouse dataset has 1.4% symmetric (equivalence + opposite) and 98.6% asymmetric (low ⇒ low + low ⇒ high + high ⇒ low + high ⇒ high) relationships. The fruit fly dataset has 12% symmetric (equivalence + opposite) and 88% asymmetric (low ⇒ low + low ⇒ high + high ⇒ low + high ⇒ high) relationships.

An interesting fact about the array technology is that alternative probesets for the same gene are not always equivalent in the network; instead, there is often a low ⇒ low relationship between them. This is consistent with previous findings of low average correlation among probesets for the same gene [7]. Boolean implications might be helpful in pointing out important differences among different probesets for the same gene, although we have not explored this issue.

### Boolean implications identify known biological properties and potentially new biological properties

**Figure 2:**
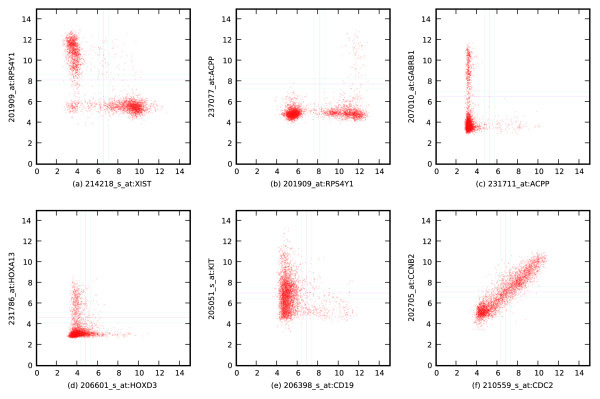
Boolean relationships follow known biology. **(a) **Gender difference, XIST high ⇒ RPS4Y1 low, male and female genes are not expressed in the same sample. **(b) **Gender tissue specific, RPS4Y1 low ⇒ ACPP low, prostate cells are from males. **(c) **Tissue difference, ACPP high ⇒ GABRB1 low, prostate and brain genes are not expressed in the same samples. **(d) **Development, HOXD3 high ⇒ HOXA13 low, anterior is different from posterior. **(e) **Differentiation, KIT high ⇒ CD19 low, differentiated B cell is different from hematopoietic stem cell. **(f) **Co-expression, CDC2 versus CCNB2.

Boolean implications capture a wide variety of currently known biological phenomena. The generated networks contain relationships that show gender differences, development, differentiation, tissue differences and co-expression, suggesting that the Boolean implication network can potentially be used as a discovery tool to synthesize new biological hypotheses. The scatter plot between XIST and RPS4Y1 in Figure 2a is an example of an asymmetric Boolean relationship that shows gender difference. RPS4Y1 is expressed only in certain male tissues because it is present solely on the Y chromosome [8], and XIST is normally expressed only in female tissues [9,10], so RPS4Y1 and XIST are rarely expressed together on the same array. Hence, there are implications RPS4Y1 high ⇒ XIST low and XIST high ⇒ RPS4Y1 low. Moreover, RPS4Y1 is Boolean equivalent to four other genes, all of which are Y-linked. Also, RPS4Y1 low ⇒ ACPP low (Figure 2b), KLK2 low, and KLK3 (PSA) low, and ACPP, KLK2, and KLK3 are all prostate-specific [11].

Boolean implications capture hierarchical relationships between tissue types. Figure 2c shows ACPP high ⇒ GABRB1 low. GABRB1 is specific to the central nervous system [12], and ACPP is prostate-specific [11]; hence, ACPP high ⇒ GABRB1 low appears sensible because the prostate is distinct from the central nervous system (CNS). On the other hand, GABRA6 is primarily expressed in the cerebellum, and we find that GABRB1 low ⇒ GABRA6 low, because the cerebellum is part of the CNS. This can be taken more literally to mean that if a sample is not part of the CNS, it is also not part of the cerebellum.

To show an example of a Boolean implication between two developmentally regulated genes, we identify HOXD3 and HOXA13 as shown in Figure 2d. HOXD3 and HOXA13 have their evolutionary origin from fruit fly antennapedia (Antp) and ultrabithorax (UBX), respectively [13]. It was recently discovered that HOXD3 and HOXA13 are expressed in human proximal and distal sites, respectively [14], a pattern of expression that is evolutionarily conserved from fruit flies. The human Boolean implication network shows that high expression of HOXD3 and HOXA13 are mutually exclusive (HOXD3 high ⇒ HOXA13 low), which is consistent with the above paper. (Unlike the findings of that paper, this relationship is not highly conserved in our analysis because orthologous mouse and fruit fly probesets for the desired genes did not have a good dynamic range in the data set.)

Implications between genes expressed during differentiation of specific tissue types also appear in the network. For example, a Boolean implication between two key marker genes from B cell differentiation, KIT and CD19, is shown in Figure 2e. KIT is a hematopoietic stem cell marker [15], and CD19 is a well-known B cell differentiation marker [16]. KIT and CD19 are rarely expressed together, as reflected by the Boolean implications CD19 high ⇒ KIT low and its contrapositive KIT high ⇒ CD19 low.

From inspecting the human network, it is clear that hundreds of genes are co-expressed that are related to the cell cycle. Two such genes, CDC2 and CCNB2, are shown in Figure 2f.

### Descriptions of data sources are consistent with the biology of the Boolean implications

We compared the Boolean implications discovered by the algorithm with the documentation of the microarray data supporting the implications. Since the hundreds of series in the Gene Expression Omnibus (GEO) are not annotated consistently, we used the descriptive web pages provided with GEO to describe each array. We developed a web interface that enabled highlighting the points in a scatter plot corresponding to arrays whose descriptive pages include a particular search term. The description pages associated with selected points in a scatter plot can be displayed. Text search of the description pages captures partial and approximate information about the microarray experiments, but it has been effective for identifying arrays associated with some particular disease and tissue types.

**Figure 3:**
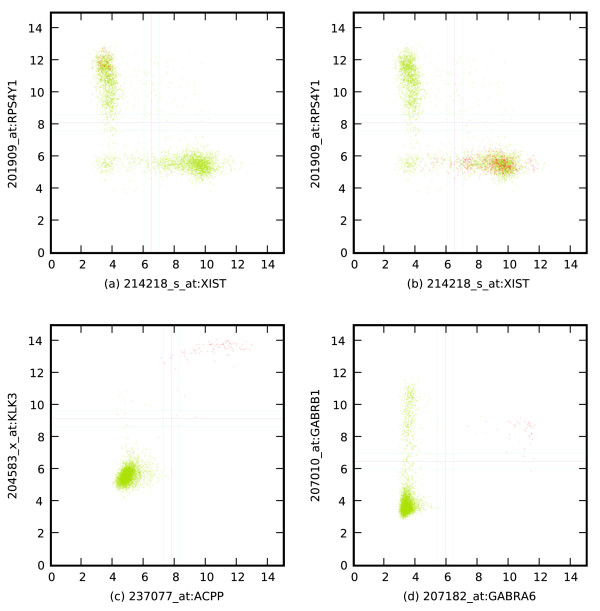
Analysis of scatter plots with various experimental conditions. Experimental conditions (highlighted as red) are determined through searching the text description of the microarray experiments. **(a) **XIST high ⇒ RPS4Y1 low, prostate microarrays are highlighted, most of them have high expression levels of RPS4Y1. **(b) **XIST high ⇒ RPS4Y1 low, breast microarrays are highlighted, most of them have high expression levels of XIST. **(c) **ACPP equivalent to KLK3, prostate microarrays are highlighted, both ACPP and KLK3 are highly expressed in prostate microarrays. **(d) **GABRA6 high ⇒ GABRB1 high, cerebellum microarrays are highlighted, GABRA6 is cerebellum-specific and GABRB1 is CNS-specific.

Figure 3a,b show the same scatter plot of RPS4Y1 versus XIST as above, but arrays are highlighted when their description pages contain the terms 'prostate' and 'breast'. As expected, all of the prostate arrays appear in the RPS4Y1 high/XIST low quadrant, and all but 6 of the 531 breast arrays appear in the RPS4Y1 low/XIST high quadrant. Inspection of the descriptions of the six breast arrays where RPS4Y1 is high reveals that four of those samples come from males, leaving only two female arrays in which RPS4Y1 has a high level of expression, possibly due to experimental error. The prostate samples come from at least three different laboratories and the breast cells come from several laboratories and include both tumor cells and cell lines.

Prostate-specific genes tend to be expressed in arrays from prostate cells. Figure 3c shows the scatter plot of ACPP versus, KLK3, highlighting the arrays whose description contains the term 'prostate'. Of 93 prostate arrays, only five have low expression of ACPP and KLK3.

Figure 3d shows a scatter plot of GABRB1 versus GABRA6 low, where GABRA6 is cerebellum-specific and GABRB1 is CNS-specific. The highlighted arrays are those whose descriptions contain the word 'cerebellum'. In these log-reduced data, the expression level of GABRA6 is 8-64 times higher in cerebellar tissue than in other cells. The arrays come from two series in GEO that contain large numbers of nervous system tissues. All of the arrays whose description contains the term 'cerebellum' have high expression levels of GABRA6. A small number of other arrays with other cell types have high expression of GABRA6, including a 'pons AB' sample, and two pilocytic cytomas. If we select the points where GABRB1 is above the threshold and examine them at random, they are almost all tissues from various parts of the brain.

### Many Boolean relationships are highly conserved across multiple species

**Figure 4:**
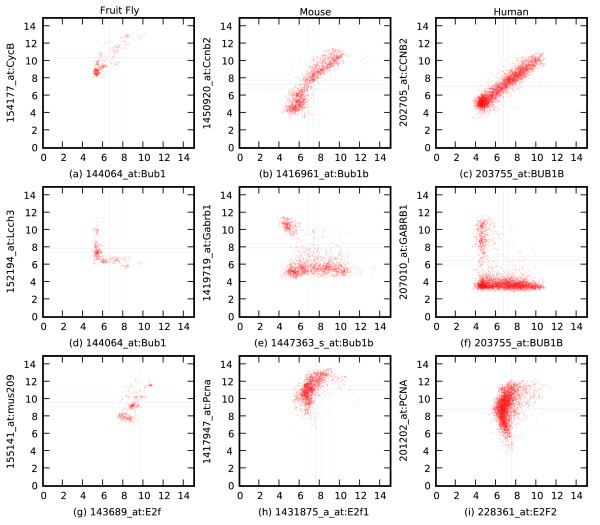
Highly conserved Boolean relationships. Orthologous CCNB2 and BUB1B equivalent relationships: **(a)** Bub1 versus CycB in fruit fly, **(b)** Bub1b versus Ccnb2 in mouse, **(c)** BUB1B versus CCNB2 in human. Orthologus BUB1B high ⇒ GABRB1 low: **(d)** Bub1 versus Lcch3 in fruit fly, **(e)** Bub1b versus Gabrb1 in mouse, **(f)** BUB1B versus GABRB1 in human. Orthologous E2F2 ⇒ PCNA high: **(g)** E2f versus mus209 in fruit fly, **(h)** E2f1 versus Pcna in mouse, **(i)** E2F2 versus PCNA in human.

We constructed a network consisting of the relationships that hold between orthologous genes in multiple species. The network of relationships that are conserved between the human and mouse networks has a total of 3.2 million Boolean implications consisting of 8,000 low ⇒ high, 2 million high ⇒ low, 0.5 million low ⇒ low, 0.5 million high ⇒ high, 10,814 equivalent and 94 opposite implications. Applying the same analysis to randomized human and mouse datasets yielded no conserved Boolean relationships, for an estimated FDR of less than 3.1 × 10^-7^. An analogous network of implications conserved across human, mouse and fruit fly has 41,260 Boolean relationships: 24,544 high ⇒ low, 8,060 low ⇒ low, 8,060 high ⇒ high and 596 equivalent and 0 opposite. The FDR for the conserved human, mouse and fruit fly Boolean implication network is less than 2.4 × 10^-5^. Figure 4 shows three examples of Boolean relationships that are conserved in humans, mice and fruit flies. The first row in Figure 4 is an example of an equivalent relationship that is conserved in all three species, and the middle and bottom rows show highly conserved high ⇒ low and high ⇒ high relationships. In the examples below, the human names are used for genes involved in conserved relationships.

The top row in Figure 4 shows that CCNB2 orthologs and BUB1B orthologs are equivalent in all three species. It is well known that both CCNB2 and BUB1B are related to the cell cycle [17,18]. The maximum connected components of the network of equivalent relationships conserved in humans, mice, and fruit flies were examined. (A maximum connected component of an undirected graph is a set of vertices for which there is a path from every vertex to every other vertex, and there are no edges from a vertex in the connected component to another connected component. In this case, the vertices represent probesets and the edges represent Boolean equivalence relationships.) The algorithm found 13 different connected components, two of which are relatively large components. The largest component has 178 genes, including well-known cell-cycle genes such as BUB1B, EZH2, CCNA2, CCNB2 and FEN1. The genes belonging to this component were analyzed using DAVID functional annotation tools [19,20] and were enriched for 'DNA replication' (2.03 × 10^-14^, 19 genes) and 'cell cycle process' (1.06 × 10^-13^, 30 genes) as significant Gene Ontology annotations. The functional annotation analysis also reported 'proteasome' and 'cell cycle' as significant Kyoto Encyclopedia of Genes and Genomes (KEGG) pathways for the largest component. The second largest component has 32 genes, and seems to be related to the nervous system with 'transport' (2.55 × 10^-8^, 16 genes) and 'synaptic transmission' (1.04 × 10^-8^, 8 genes) as significant Gene Ontology annotations. This component is enriched for calcium signaling pathway in the KEGG database. The list of genes for the components and the DAVID functional annotation results are included in Additional data files 2-6.

The connected components described above have biologically meaningful relationships. CCNB2 and BUB1B play roles in mitosis [18,21], EZH2 is a histone methyltransferase [22], CCNA2 is required for G1/S transition [23] and FEN1 has endonuclease activity during DNA repair [24]. Surprisingly, all these genes are highly correlated in all three species. Interestingly, of the two human homologs of *Drosophila *polycomb-group gene *Enhancer-of-zeste *(E(z)), EZH1 and EZH2, only EZH2 maintains a functional association with other cell cycle genes. EZH1 might have evolved to acquire a different function than EZH2 in mammals. In addition, there are highly conserved equivalent genes that are part of the same protein complexes, such as CDC2-CCNB2, EED-EZH2, RELB-NFKB2, RFC1-RFC2-RFC4, and MSH2-MSH6. There is also a conserved cluster of four genes - NDUFV1, IDH3B, CYC1 and UQCRC1 - that are all related to generation of energy through oxidative phosphorylation and the electron transport chain.

The middle row in Figure 4 shows an asymmetric relationship that is conserved in all three species: BUB1B high ⇒ GABRB1 low. GABRB1 is a receptor for an inhibitory neurotransmitter in vertebrate brains [25]. Inspection of the descriptions of arrays in which orthologs of GABRB1 are expressed shows that they are overwhelmingly from CNS tissue in humans and mice and 'brain' or 'head' samples from fruit flies. It is surprising to see that the Boolean implication between GABRB1 and BUB1B is conserved in vertebrates and fruit flies. This relationship suggests that cells expressing the GABRB1 neurotransmitter are less likely to be proliferating. The bottom row in Figure 4 shows an asymmetric relationship between two well-known cell cycle regulators, E2F2 and PCNA [26-28].

**Figure 5:**
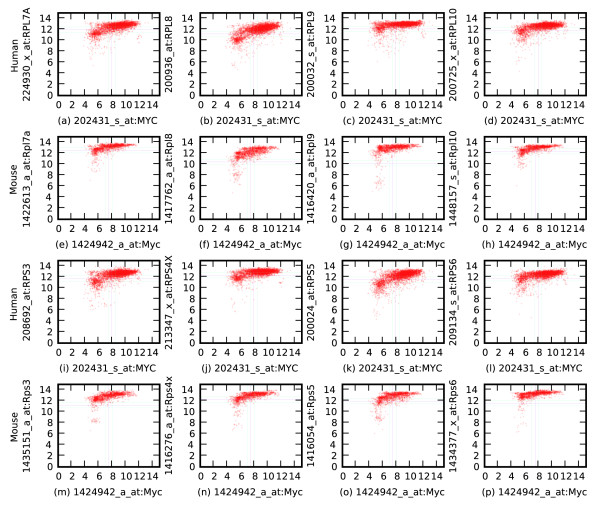
Conserved Boolean relationships between MYC and ribosomal genes. **(a-h) **The scatterplots show Boolean relationships between MYC and a few selected genes for large ribosomal subunits in both human and mouse datasets. **(i-p) **Boolean relationships between MYC and few selected ribosomal small subunit genes in both human and mouse datasets. (a-d, i-l) Human datasets. (e-h, m-p) Mouse datasets. (a) MYC high ⇒ RPL7a. (b) MYC high ⇒ RPL8 high. (c) MYC high ⇒ RPL9 high. (d) MYC high ⇒ RPL10 high. (e) Myc high ⇒ Rpl7a. (f) Myc high ⇒ Rpl8 high. (g) Myc high ⇒ Rpl9 high. (h) Myc high ⇒ Rpl10 high. (i) MYC high ⇒ RPS3. (j) MYC high ⇒ RPS4X high. (k) MYC high ⇒ RPS5 high. (l) MYC high ⇒ RPS6 high. (m) Myc high ⇒ Rps3. (n) Myc high ⇒ Rps4x high. (o) Myc high ⇒ Rps5 high. (p) Myc high ⇒ Rps6 high.

Figure 5 shows the Boolean implications between MYC and ribosomal genes in the network of relationships that are conserved between humans and mice. The implication is MYC high ⇒ ribosomal genes high for both large and small ribosomal subunits. This implication is consistently observed for 19 genes for large subunits of the ribosome (*p*-value <3 × 10^-26^) and 15 genes for small subunits of the ribosome (*p*-value <1 × 10^-22^). MYC has been shown to regulate ribosomal genes in a recently comparative study between human and mouse [29]. In this study, the high expression levels of MYC and ribosomal genes in human lymphoma were compared with the gene signature associated with MYC-induced tumorigenesis in mice.

### Boolean implication networks are more comprehensive than correlation-based networks

To compare the properties of Boolean implication networks to correlation-based networks, both types of networks were constructed based on human CD (Cluster of differentiation) antigen genes. This set of genes was chosen because it is a relatively small and coherent subset of biologically interesting genes, and a correlation network can be constructed more rapidly than if all the probesets on the arrays were used, which would have taken an unreasonable amount of computation. The correlation-based network on human CD genes was computed as described in Materials and methods.

**Figure 6:**
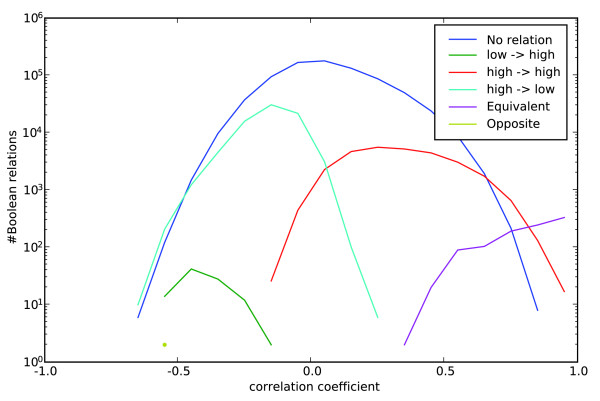
Comparison of Boolean implications with correlation. On human CD (clusters of differentiation) genes, this plot shows the histogram of different types of Boolean relationships. Blue, no relationships; green, low ⇒ high; red, high ⇒ high; cyan, high ⇒ low; magenta, equivalent; yellow, opposite.

Figure 6 shows histograms of the various kinds of Boolean relationships with respect to the Pearson's correlation coefficients between expression levels of the same pairs of genes. As expected, highly correlated genes generally correspond to symmetric Boolean relationships; 80% of the symmetric Boolean relationships have correlation coefficients more than 0.65. Figure 6 shows that the number of Boolean equivalent pairs increases linearly with the correlation coefficient, suggesting that most of the Boolean equivalence have good correlation coefficients. Therefore, gene pairs with high correlation coefficients are almost always Boolean equivalent.

On the other hand, asymmetric Boolean relationships usually display poor correlation; 98.8% of the asymmetric Boolean relationships on the human CD genes have correlation coefficients ranging from -0.65 to 0.65 (correlation-based networks are often based on gene pairs having a threshold of 0.7 or greater for the correlation coefficient [3,4,30]). The histograms in Figure 6 suggest that it would be very difficult to find approximately the same asymmetric relationships using a filter based on correlation coefficients, because the number of non-relationships in a given range of correlation coefficients usually greatly exceeds the number of asymmetric relationships.

### Boolean implication networks are not scale free

**Figure 7:**
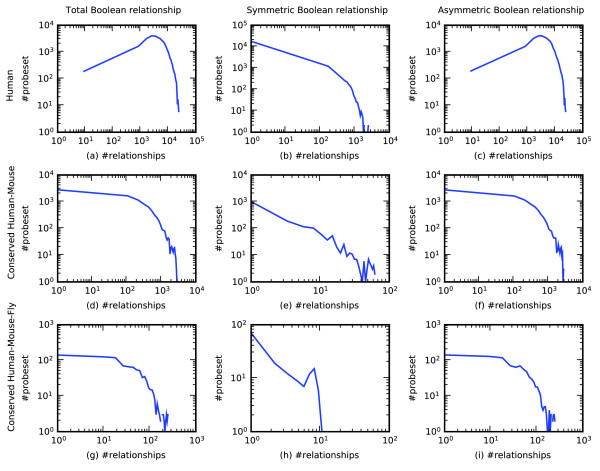
Properties of Boolean implication network. Log-log plot of the histogram of the probesets with respect to their number of Boolean relationships. Human Boolean network: **(a) **total, **(b) **symmetric, and **(c) **asymmetric Boolean relationships. Conserved human and mouse Boolean network: **(d) **total, **(e) **symmetric, **(f) **asymmetric Boolean relationships. Conserved human, mouse and fruit fly Boolean network: **(g) **total, **(h) **symmetric, **(i) **asymmetric Boolean relationships.

It has often been observed that other biological networks are scale-free [31-36]. To study the global properties of Boolean implication networks, we plotted the frequency of the probesets against their degree as shown in Figure 7. (The degree of a probeset is the number of Boolean relationships involving that probeset.) Each log-log plot shows the degree on the horizontal axis and the number of probesets with that degree on the vertical axis. The top row in Figure 7 corresponds to the human Boolean implication network. From left to right are shown the total Boolean relationships, symmetric Boolean relationships alone, and asymmetric Boolean relationships alone. These plots are comparable to the Boolean implication networks for mice and fruit flies (Figure S1 in Additional data file 1). The middle row in Figure 7 corresponds to the conserved Boolean implication network between humans and mice. Finally, the bottom row in Figure 7 shows the conserved Boolean implication network between humans, mice and fruit flies. As can be seen from the figures, the plots for symmetric Boolean relationships (second and third columns in Figure 7) are close to linear. However, the plots for total Boolean relationships (first column in Figure 7) are non-linear. Therefore, the overall Boolean implication network is not scale free.

### Computing the Boolean implication network is fast and the output is transparent

The total computation time to construct the network of implications for the human dataset was 2.5 hours on a 2.4 Ghz computer with 8 GB of memory. The human dataset consisted of 54,677 distinct probesets from 4,787 microarrays. The computation time for the mouse dataset was 1.6 hours. This data set has 45,101 probesets and 2,154 microarrays. Finally, the computation time for the fruit fly dataset, consisting of 14,010 probesets and 450 microarrays, was 2 minutes.

Generating the Boolean implication network is conceptually a simple process. The relationships are immediately evident upon inspection of a scatter plot of the data points of expression levels for the two related genes, and are thus completely transparent and intuitive to biologists, unlike some approaches that find complex relationships that can be more difficult for users to interpret.

### Related work

There has been no previous published attempt to discover Boolean implications for the full genome on large-scale gene expression data. Most previous work on extracting networks from large amounts of expression data has focused on finding pairs of co-expressed genes, based on correlation or measures of mutual information [1-6,37-41]. Our method generally finds the same kinds of relationships by identifying Boolean equivalent or opposite gene pairs, which correspond well with genes that are strongly positively or negatively correlated. If we were to consider only the network of Boolean equivalent and opposite relationships, it would still be one of the largest co-expression networks constructed to date (and it would be based on a larger quantity of data than other networks); however, the full network of asymmetric relationships is about 100 times larger than that.

Various methods for finding implications of various kinds have been used for other types of data. In the field of psychology, Boolean implications between answers to questions on questionnaires have been proposed to capture structure in knowledge and attitudes (for example, if a student can solve a problem of type X, then he/she can solve a problem of type Y) [42,43]. In the literature, these methods have been applied to data with very small numbers of variables, so it is difficult to understand they could work on a scale of tens of thousands of genes. Some of the discovery methods are based on extremely inefficient algorithms, such as enumerating all possible quasi-orders over the variables to find the one that best fits the data. These methods also assume that the data are already binary, so it was not necessary to discover thresholds as we have done or deal with data that are very close to the thresholds.

Nested effects models, which look for subset relationships in response to perturbations, have been applied to high-throughput data, including gene expression data to infer the structure of pathways [44,45]. For example, if the genes affected by interfering with gene A are a subset of the genes affected by interfering with gene B, we might conclude that A is downstream of B. This method could, in theory, be adapted to our problem, since subset relationships are implications (*S *⊆ *T *can be interpreted as '*x *∈ *S *⇒ *x *∈ *T*'). However, this work goes to significant effort to ensure that the discovered relationships are (usually) transitive, requiring triples of genes to be considered, so that it scales to 'dozens of genes'. Another method for discovering Boolean implication networks somewhat similar to ours was described for probabilistic reasoning [46], but it seems that particular method has not previously been applied to microarray data - or indeed, to any aspect of biology.

Several techniques have been proposed for building more sophisticated and complex network models from high-throughput data. The basic problem with these approaches is that they need to consider an infeasible number of candidate network models, so the methods cannot scale to thousands of variables. Additionally, there are usually many models that fit the data well, so it is difficult to know which model to choose. On the other hand, Boolean implications report invariant relationships in the data, which are limited in expressiveness but relatively easy to confirm by inspection, and the methods for computing them scale to the whole genome. More specifically, Bayesian networks are frequently constructed to find relationships among variables in high-throughput data [47-54]. This requires learning the structure of the networks, which is a problem of super-exponential computational complexity. Although heuristics and approximations are available to improve the efficiency of these procedures, they cannot be applied to systems of more than a few dozen variables. Generalizations of Bayesian networks, such as graphical Gaussian models [55,56], have similar issues. The resulting Bayesian networks associate a random variable *X *with a set of parent variables, where the probability of *X *conditioned on its parents is independent of the probabilities of all non-descendent variables given the values of its parent variables. Although Boolean implications could be inferred in some cases from the joint probability distributions of each node and its parents, the relationship between Bayesian networks and Boolean implication networks is not obvious.

Boolean circuit models are another type of complex network that has been extracted from high-throughput data [57-60]. A Boolean circuit is a network of logic gates whose inputs and outputs represent concentrations of proteins, up- or down-regulation of genes, and so on (in some cases, the models have more than two values, but the basic methods are similar). As with Bayesian networks, the number of circuits explodes with the number of variables, so these methods do not scale to the full genome, and many different models may match the same data. It might be possible to apply some of the same techniques to find implication relationships, but there are no published reports of that having been done.

Gene expression relationships that are conserved across multiple species have been used to infer likely regulatory relationships [30,61-67]. This work has not examined conserved asymmetric relationships. It is easy to perform conservation analysis on Boolean implication networks, which involves checking if the orthologous gene pairs have the same Boolean relationships, while other approaches require non-trivial probabilistic measures of conservation. Numerous studies construct conserved gene-interaction networks across several species using correlated genes. An early study of this type improved the accuracy of predicting functional gene interactions by using conserved co-expression between *Saccharomyces cerevisiae *and *Caenorhabditis elegans *[67]. They used a correlation coefficient threshold of 0.6. Subsequently, another study identified 22,163 gene pairs from 3,182 DNA microarrays from humans, flies, worms and yeast [64]. This study used a rank order statistic to compute a probabilistic measure of the conserved co-expression in multiple species. Further, Bayesian analysis was combined with conservation to build gene networks for yeast and human using cell cycle data [65]. Later studies focused on human and mouse to discover conserved gene expression in brain [63] and gametogenesis [61].

## Conclusion

Boolean implications provide a perspective on genome-scale data that reveals biologically meaningful relationships that are missed by other types of analysis, either because those methods search for different types of relationships, or because they do not scale to the whole genome level. A meta-analysis of thousands of arrays for three different species shows some of the potential of Boolean implications for exposing biological information in data. The collection of all implication relationships is a network. In the networks of implications constructed in the meta-analysis, there are almost 100 times as many implication relationships as equivalences. Differences associated with gender and tissue-type are readily apparent. Relationships between genes that are active only during specific developmental or differentiation stages are also evident. Many Boolean relationships are conserved across humans, mice and fruit flies. There are highly conserved relationships among clusters of genes that are enriched with the cell cycle- and CNS-specific genes. The conserved asymmetric Boolean implications between MYC and ribosomal genes suggest the presence of biologically relevant regulatory relationships in the implication network. The Boolean implication network could conceivably offer a new discovery platform, providing new biological hypotheses to be further explored experimentally. The networks can be computed rapidly even using massive amounts of gene expression data, and the output is transparent and easy to navigate. The Boolean network is available for exploration at the BooleanNet website [68].

It is important to understand the limitations of Boolean implications. Each implication is an empirically observed relationship in the data, which may not hold for data gathered for different tissue types or under different conditions. Like correlation networks, Boolean implication networks do not capture causality. Indeed, known regulatory relationships between transcription factors and their targets often do not have corresponding implications. This is to be expected, since there are many other factors involved in gene regulation that are not apparent in gene expression data, such as protein activation, participation in complexes involving several proteins, and combinatorial regulation on promoters.

We believe the greatest potential of Boolean implications is in combination with other types of data and other types of analysis. For example, in combination with data from particular perturbations, such as gene silencing or drug treatment, and in conjunction with transcription factor binding relationships, some implications could be interpreted as causal relationships. Furthermore, implications can be used to constrain the search for more complex models. For example, the Boolean relationship 'C is high only when A is high and B is low' can hold only if the implications 'A low ⇒ C low' and 'B high ⇒ C low' hold.

## Materials and methods

### Data collection and preprocessing

**Figure 8:**
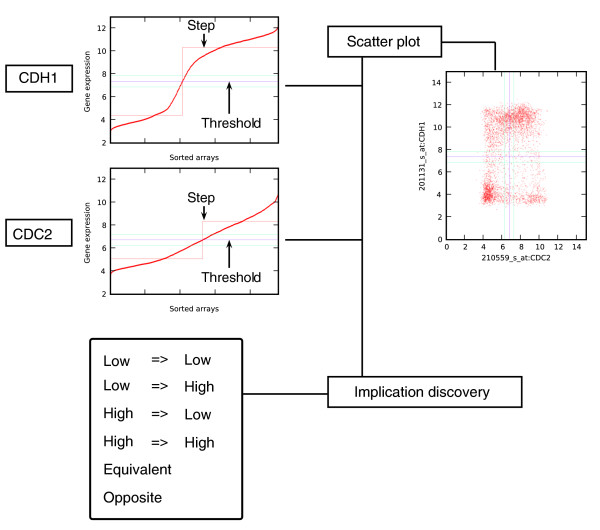
Boolean implication extraction process. The expression levels of each probeset are sorted and a step function fitted (using StepMiner) to the sorted expression level w minimizes the square error between the original and the fitted values. A threshold *t *is chosen, where the step crosses the original data. The region between *t *- 0.5 and *t *+ 0.5 is classified as 'intermediate', the region below *t *- 0.5 is classified as 'low' and the region above *t *+ 0.5 is classified as 'high'. The examples show probesets for two genes, CDH1 and CDC2. As can be seen, CDH1 has a sharp rise between 6 and 9 and the StepMiner algorithm was able to assign a threshold in this region. CDC2, however, is very linear, and the StepMiner algorithm assigns the threshold approximately in the middle of the line. A scatter plot is shown to illustrate the analysis. Each point in the scatter plot corresponds to a microarray experiment, where the value for the x-axis is CDC2 expression and the value for the y-axis is CDH1 expression. Boolean implication discovery analysis is performed on a pair of probesets, which ignores all the points that lie in the intermediate region and analyzes the four quadrants of the scatter plot. Four asymmetric relationships (low ⇒ low, low ⇒ high, high ⇒ low, high ⇒ high) are discovered, each corresponding to exactly one sparse quadrant in the scatter plot; and two symmetric relationships (equivalent and opposite) are discovered, each corresponding to two diagonally opposite sparse quadrants.

CEL files for 4,787 Affymetrix U133Plus 2.0 human microarrays, 2,154 Affymetrix 430 2.0 mouse arrays, and 450 Affymetrix Genome 1.0 *Drosophila *arrays were downloaded from NCBI's GEO [69]. These array types were chosen because they are widely used, and because results from different arrays can be compared more easily than results from two-channel arrays. The datasets were normalized using the standard Robust multi-chip analysis algorithm (RMA) [70]; however, the available version of RMA uses excessive amounts of primary memory when normalizing thousands of arrays, so the program was re-written to increase memory efficiency. Boolean expression levels were assigned for each gene in each array, using the log (base 2) of the expression values (Figure 8 illustrates this process).

First, a threshold was assigned to each gene using the StepMiner algorithm [71], which was originally designed to fit step functions to time-course data. For this application, the expression values for each gene were ordered from low-to-high, and StepMiner was used to fit a rising step function to the data that minimizes the differences between the fitted and measured values. This approach places the step at the largest jump from low values to high values (but only if there are sufficiently many expression values on each side of the jump to provide evidence that the jump is not due to noise), and sets the threshold at the point where the step crosses that original data (as shown in Figure 8). In the case where the gene expression levels are evenly distributed from low to high, the threshold tends to be near the mean expression level.

If the assigned threshold for a gene is *t*, expression levels above *t + *0.5 are classified as 'high', expression levels below *t *- 0.5 are classified as 'low', and values between *t *- 0.5 and *t *+ 0.5 are classified as 'intermediate'. Points in the intermediate region are ignored, because they are much more likely to appear on the wrong side of the threshold due to noise. The choice of the interval width is based on an estimate of the minimum noise in the gene expression based on the gene whose standard deviation is at the 5th percentile from the bottom (that is, looking at the standard deviations of genes that have nearly constant expression levels over all the arrays). The standard deviation is a little less than 0.26, so ± 0.5 is almost exactly two standard deviations from the threshold. This is consistent with our observation that the ratios of the most tightly correlated genes still vary by a factor of two.

Finally, whenever more than two-thirds of the expression values of a gene were at an intermediate level of expression, the gene was excluded from further analysis, due to insufficient dynamic range in the expression values.

### Discovery of Boolean relationships

All pairs of features with sufficient dynamic range were analyzed to discover potential Boolean relationships. There are six possible Boolean relationships between genes A and B that are constructed from four possible Boolean implications: A low ⇒ B low, A low ⇒ B high, A high ⇒ B low, and A high ⇒ B high. Each of the above implications is detected by checking whether one of the four quadrants in the scatter plot of Figure 8 is significantly sparsely populated with points compared with the other quadrants (intermediate values for A and B are ignored in this analysis). There are at most two possible sparse quadrants because the thresholds always separate a reasonable number of low and high expression levels for each gene. Each sparse quadrant corresponds to an implication. If A high ⇒ B high and A low ⇒ B low, A and B are considered to have equivalent levels of Boolean expression. When A high ⇒ B low and A low ⇒ B high, A and B are considered to have an opposite Boolean relationship. In both of these cases, two diagonally opposite quadrants are significantly sparse. In other cases, where there is only one sparse quadrant, the Boolean relationships between A and B have the same name as Boolean implications: A low ⇒ B low, A low ⇒ B high, A high ⇒ B low, and A high ⇒ B high. There are two tests that must succeed for the relationship between A and B to be considered an implication. For concreteness, let us consider whether the low-low quadrant is sparse, yielding an implication A low ⇒ B high. First, the number of expression values in the sparse quadrant must be significantly less than the number that would be expected under an independence model, given the relative distribution of low and high values for A and B. Specifically, if *a*_00_, *a*_01_, *a*_10_, *a*_11 _are the number of expression values where A and B are low and low, low and high, high and low, and high and high, respectively, a threshold on the following statistic is performed to test whether the low-low quadrant is sparse.

*total *= *a*_00 _+ *a*_01 _+ *a*_10 _+ *a*_11_

number of A low expression values = *nA*_*low *_= (*a*_00 _+ *a*_01_)

number of B low expression values = *nB*_*low *_= (*a*_00 _+ *a*_10_)

*expected *= (*nA*_*low*_/total * nB_*low*_/*total*) * *total *= (*a*_00 _+ *a*_01_) * (*a*_00 _+ *a*_10_)/*total*

*observed *= *a*_00_

$$statistic=\frac{\left(expected-observed\right)}{\sqrt{expected}}$$

Second, the observed values in the sparse quadrant are considered erroneous points and a sparse quadrant must have a small number of erroneous points. A maximum likelihood estimate of the error rate is computed as follows:

$$error\;rate=\frac{1}{2}\left(\frac{a_{00}}{\left(a_{00}\_a_{01}\right)}+\frac{a_{00}}{\left(a_{00}+a_{10}\right)}\right)$$

A second threshold on this error rate is performed to ensure that the quadrant is truly sparse. If the above tests succeed, the low-low quadrant is considered sparse and, therefore, A low ⇒ B high is inferred. An implication is considered significant if the first statistic is greater than 3.0 and the error rate is less than 0.1.

### Computation of false discovery rate

Given the large number of probesets and even larger number of potential relationships, it is necessary to evaluate the significance of the relationships discovered by the above algorithm. To this end, we computed a FDR for each network by randomly permuting the expression values for each gene independently [72], and then extracting the Boolean implication network as above (using thresholds). This analysis was repeated 20 times to compute the average number of Boolean relationships in the randomized data. The FDR is the ratio of the average number of Boolean relationships in the randomized data to the original data.

### Correlation network for human CD genes

Human CD genes were selected for comparison against a correlation-based network. The set of genes includes 966 Affymetrix U133 Plus 2.0 human probesets. Pearson's correlation coefficients for all 466,095 pairs of genes were computed. Boolean implications were extracted from these data, as above, to compare the Boolean implication network with the correlation-based network.

### Discovery of conserved Boolean relationships

Mouse and fruit fly orthologs for human genes were selected from the EUGene database [73]. For each Boolean relationship in the human dataset, a conserved relationship was detected if any of the mouse orthologs of the first human gene had a significant Boolean relationship with any other mouse ortholog of the second human gene. To find conserved Boolean relationships in all three species, we checked if any of the fruit fly orthologs of the first mouse gene had a significant Boolean relationship with any other fruit fly orthologs of the second mouse gene for each conserved relationship in human and mouse.

### Connected component analysis

For the maximum connected component analysis, an undirected graph was built with the gene names as nodes and the edges from Boolean equivalent relationships. Initially, each distinct node was considered to be a connected component, and small connected components were merged repeatedly using a standard union-find algorithm [74] until there were no more edges connecting distinct components.

## Abbreviations

CNS: central nervous system; GEO: Gene Expression Omnibus; FDR: false discovery rate; KEGG: Kyoto Encyclopedia of Genes and Genomes.

## Authors' contributions

DS and DLD conceived of discovering Boolean implications. DS designed and implemented BooleanNet, and drafted the manuscript. DLD participated in the design, implementation and writing. RT helped with the statistical test for Boolean implication. SKP helped conceptualize the direction of the project and participated in writing. AJG participated in the discussions on Boolean implication discovery and drafted the scale free section of the manuscript.

## Additional data files

The following additional data are available with the online version of this paper. Additional data file 1 is a log-log plot of the histogram of probesets with respect to their number of Boolean implications in the human, mouse and fruit fly datasets. Additional data file 2 is a list of connected components, where each line is a tab separated HUGO gene symbol name. Additional data file 3 lists the results of GO analysis on the largest cluster. Additional data file 4 lists the results of GO analysis on the second largest cluster. Additional data file 5 is DAVID functional annotation analysis using KEGG pathway and on the largest cluster. Additional data file 6 is DAVID functional annotation analysis using KEGG pathway and on the second largest cluster.

## Supplementary Material

Additional data file 1 Log-log plot of the histogram of probesets with respect to their number of Boolean implications in the human, mouse and fruit fly datasets. Click here for file

Additional data file 2 Each line is a tab separated HUGO gene symbol name. Click here for file

Additional data file 3 GO analysis on the largest cluster. Click here for file

Additional data file 4 GO analysis on the second largest cluster. Click here for file

Additional data file 5 DAVID functional annotation analysis using KEGG pathway and on the largest cluster. Click here for file

Additional data file 6 DAVID functional annotation analysis using KEGG pathways and on the second largest cluster. Click here for file
