# Supplementary material for: Boolean implication networks derived from large scale, whole genome microarray datasets
Source: Genome Biol. 2008 Oct 30;9(10):R157. doi: 10.1186/gb-2008-9-10-r157 (PMC2760884; doi:10.1186/gb-2008-9-10-r157)
Supplement: Additional data file 1 — Log-log plot of the histogram of probesets with respect to their number of Boolean implications in the human, mouse and fruit fly datasets. [file gb-2008-9-10-r157-S1.doc]

### Supplementary information

**Figure S1. Properties of human mouse and fruit fly Boolean implication networks**: log-log plot of the histogram of the probesets with respect to their number of Boolean relationships. Human Boolean network: (a) Total, (b) symmetric, (c) asymmetric Boolean relationships. Mouse Boolean network: (d) Total, (e) symmetric, (f) asymmetric Boolean relationships. Fruit fly Boolean network: (g) Total, (h) symmetric, (i) asymmetric Boolean relationships.


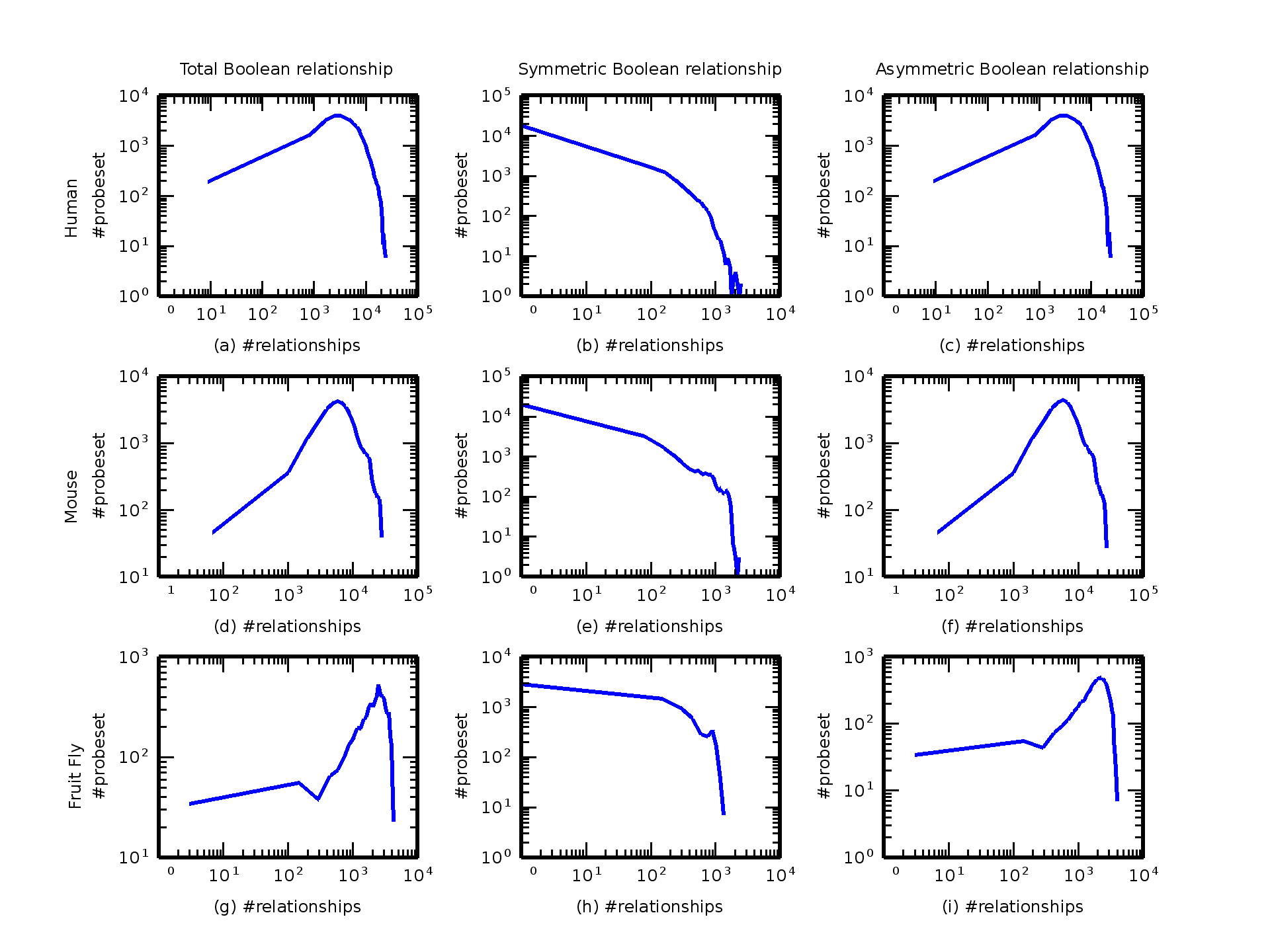


The following files can be accessed at the BooleanNet website [68].

**File 2. Connected component analysis**: The cluster of genes can be found in each line as tab separated HUGO gene symbol name.

**File 3. DAVID functional annotation (GO Analysis) on the largest cluster**

**File 4. DAVID functional annotation (GO Analysis) on the second largest cluster**

**File 5. DAVID functional annotation (KEGG) on the largest cluster**

**File 6. DAVID functional annotation (KEGG) on the second largest cluster**
